# Leukocytosis in Cushing’s syndrome persists post-surgical remission and could predict a lower remission prognosis in patients with Cushing’s disease

**DOI:** 10.1007/s40618-025-02535-2

**Published:** 2025-01-28

**Authors:** Hiba Masri-Iraqi, Yaron Rudman, Tzipora Shochat, Shiri Kushnir, Ilan Shimon, Maria Fleseriu, Amit Akirov

**Affiliations:** 1 Institute of Endocrinology, Beilinson Hospital, Rabin Medical Center, Petach Tikva, 49100 Israel; 2 Faculty of Medicine, Tel Aviv University, Tel Aviv, Israel; 3 Biostatistics Unit, Rabin Medical Center, Beilinson Hospital, Petah Tikva, Israel; 4 Research Authority, Rabin Medical Center, Beilinson Hospital, Petah Tikva, Israel; 5 Pituitary Center, Departments of Medicine and Neurological Surgery, Oregon Health & Science University, Portland, OR USA

**Keywords:** Cushing’s syndrome, Pituitary, White Blood Cell

## Abstract

**Context:**

Leukocytosis frequently noted in Cushing’s syndrome (CS), along with other blood cell changes caused by direct and indirect cortisol effects.

**Objective:**

Assess baseline white blood cell (WBC) profile in CS patients compared to controls and WBC changes pre- and post-remission after surgical treatment for CS.

**Design:**

A comparative nationwide retrospective cohort study.

**Setting:**

Data from Clalit Health Services database.

**Patients:**

297 patients (mean age 51 ± 16.1 years, 73.0% women) with CS and 997 age-, sex-, body mass index-, and socioeconomic status-individually matched controls. Ectopic CS or adrenal cancer patients were excluded.

**Main outcome measure:**

Mean WBC, neutrophils, and neutrophil-to-lymphocyte ratio (NLR) two-years before and after pituitary or adrenal surgery. WBC and neutrophils are expressed as Kcells/µl.

**Results:**

At baseline, leukocytosis was observed in 21.5% of patients with CS vs. 8.9% of controls (*P* < 0.001). Patients with CS had significantly higher WBC (8.8 ± 2.88 vs. 7.54 ± 2.45, *p* < 0.0001), neutrophils (5.82 ± 2.38 vs. 4.48 ± 1.97, *p* < 0.0001), and NLR (3.37 ± 2.63 vs. 2.27 ± 1.86, *p* < 0.0001) compared to controls, regardless of pituitary or adrenal source of hypercortisolemia. Post-surgery, patients with CS experienced significant decreases in mean WBC (-0.57 ± 2.56, *p* < 0.0001), neutrophils (-0.84 ± 2.55, *p* < 0.0001), and NLR (-0.63 ± 2.7, *p* < 0.0001). Despite achieving disease remission, patients with CS still had higher WBC (8.11 ± 2.4 vs. 7.46 ± 2.17, *p* = 0.0004) and neutrophils (4.71 ± 2.10 vs. 4.41 ± 1.87, *p* = 0.03) compared to controls. Patients with CD and baseline leukocytosis had lower remission rate than those with normal WBC (36.7% vs. 63.9%, *p* = 0.01).

**Conclusions:**

At diagnosis, CS patients have elevated WBC, neutrophils, and NLR compared to controls. Remission does not normalize WBC levels in all patients, and baseline leukocytosis predicts a poorer remission prognosis in CD.

## Introduction

Cushing syndrome (CS) is characterized by hypercortisolism, typically resulting from ACTH pituitary hypersecretion, adrenal cortisol excess and in fewer cases, ectopic ACTH secretion [1–4]. The clinical features, not present in all patients with the same penetrance include several more specific ones: proximal myopathy, facial plethora, round face, dorsocervical fat pads, purple striae, easy bruising, thin skin and fragility fractures, as well as several comorbidities central obesity, diabetes mellitus, hypertension, osteoporosis, susceptibility to infections, and others [3–5]. Over the years, various scores were developed to assess the probability of a CS diagnosis based on clinical presentation and initial biochemical work-up [6–8].

Alterations in blood cell count have been observed in patients with endogenous and exogenous: white blood cells count elevation, neutrophilia and lymphopenia [9–12]. In recent years, the neutrophil to lymphocyte ratio (NLR), one of the inflammation-based scores has emerged as a significant marker in various conditions including in patients with CS and has been proposed as an indicator of cortisol excess in adrenal adenomas [10, 13, 14].

We have previously reported in a small cohort of patients with Cushing’s disease(CD) changes in WBC count, although NLR was not investigated [12]. In this larger cohort of patients with multiple causes of CS we present details on WBC count at diagnosis of hypercortisolism and subsequent changes upon achieving remission.

## Methods

This retrospective case control study is based on Clalit Health Service (CHS) electronic database, the public healthcare system serving approximately 4.5 million people in Israel with clinical data retrieved from outpatients’ clinics and hospitals.

**Fig. 1:**
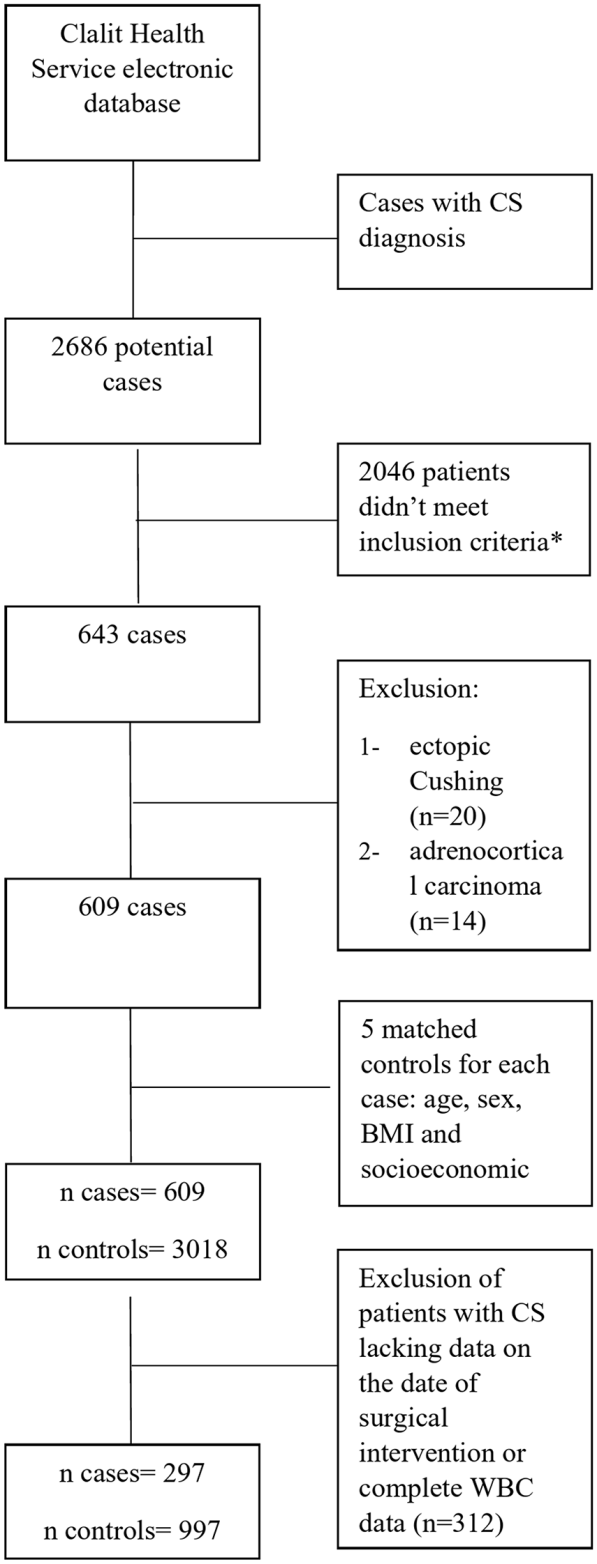
Algorithm of patients selection from Clalit health services. *Inclusion criteria: (i) recorded 24-hour urinary free cortisol (UFC) > 4 × upper limit of normal (ULN) confirmed (ii) 24-hour UFC > 3 × ULN and a surgical intervention to remove the pituitary or adrenal adenoma

The recruitment of the patients with CS from the CHS electronic database has been previously described [15]. Cases with the initial recorded diagnosis of CS were further investigated, and for this study diagnosis of CS was confirmed if they met one of the following criteria: (i) any recorded 24-hour urinary free cortisol (UFC) > 4 × upper limit of normal (ULN) confirmed and (ii) 24-hour UFC > 3 × ULN and a surgical intervention to remove the pituitary or adrenal adenoma. Each case was confirmed by reviewing the data by the authors (AA, YR); cases suspected to be ectopic CS or adrenocortical carcinoma were excluded. Each case of Cushing syndrome was initially matched with five controls from the general population based on age, sex, BMI, and socioeconomic status. These controls were randomly selected and had no history of investigations for hypercortisolism. For this analysis, CS patients lacking data on the date of surgery were excluded, along with their corresponding matched controls (Fig. 1).

Data collected included age, sex, body mass index (BMI), socioeconomic status, smoking status, comorbidities at diagnosis such as type 2 diabetes mellitus, hypertension, dyslipidemia, ischemic heart disease, stroke, cardiovascular disease, chronic kidney disease and malignancy. Laboratory data extracted relevant to our study included: WBC, neutrophils and lymphocytes count. These laboratory parameters were also used to calculate the NLR. These values were recorded several times within the 24 months prior to diagnosis and within the 24 months following diagnosis, and means were calculated from multiple data points.

Biochemical remission was defined as achieving a 24-hour UFC level within the normal range without the need for any medical interventions to manage hypercortisolism (such as metyrapone, ketoconazole, osilodrostat, cabergoline, or pasireotide), or requiring glucocorticoid replacement for hypocortisolism following pituitary or adrenal surgery.

The follow-up period was determined from the time of surgical intervention to treat CS for patients with CS, and for their individually matched controls it began at the age of each case’s intervention, and continued until death, termination of CHS membership, or until the date of data collection, June 30, 2023.

The primary outcome was to ascertain how blood parameters, including WBC, neutrophils, lymphocytes and NLR were affected in CS patients compared to controls, and determine how they changed with disease remission within two years of surgical intervention. Secondarily, we analyzed how differences in CS etiology affected the WBC profile and correlation of WBC at baseline with remission prognosis.

### Statistical analysis

The statistical analysis for this paper was generated using SAS Software, Version 9.4.

Continuous variables were presented by mean ± standard deviation or median and IQR, Categorical variables were presented by (N,%). Normality of continuous variables was assessed using the Kolmogorov-Smirnov test. T-Test was used to compare the value of normally distributed continuous variables between study groups, Wilcoxon was used for non-normal continuous variables and Fisher’s exact test was used to compare the value of categorical variables between study groups. Logistic regression analysis was performed to evaluate the association between leukocytosis at diagnosis and remission status after two years. Two-sided p values less than 0.05 were considered statistically significant.

## Results

The initial search in CHS database identified 609 patients with CS. Following exclusion of cases that did not meet this study criteria, complete data relevant to this study was available for 297 patients (73% women, mean age ± SD, 51 ± 16.1 years) and 997 controls, selected initially based on age, sex, BMI and socioeconomic status (Fig. 1). Patients with CS were three years older (*p* = 0.0028) and more frequently diagnosed with hypertension (*p* < 0.001) but other comorbidities did not differ significantly from the controls (Table 1).

**Table 1:** Baseline characteristics

	Controls	CS	*p*-value
**N**	997	297	
**Age**,** years** mean (SD)	54.1(16.28)	51 (16.09)	0.0028
**Follow-up**,** months (SD)**	128.6(61.6)	132.4(62.7)	0.15
**BMI**	30.6(6.5)	30.9(7.1)	0.91

	**N**	**%**	**N**	**%**	*p*-value
**Gender**					0.19
Male	235	23.5	81	27.2	
Female	762	76.4	216	72.7	
**Socioeconomic status**					0.63
Low	113	12.1	30	10.9	
Medium	552	59.4	172	62.7	
High	264	28.4	72	26.2	
**Smoking status**					0.37
Never	452	62.8	128	61.2	
Past smoker	145	20.14	37	17.7	
Current smoker	132	17.08	44	21.05	
**Comorbidities at diagnosis**					
Diabetes mellitus	230	23.07	80	26.9	0.18
Hypertension	514	51.5	188	63.3	< 0.001
Dyslipidemia	496	49.7	153	51.5	0.5
Ischemic heart disease	114	11.4	45	5.15	0.08
Stroke	53	5.32	18	6.06	0.66
Cardiovascular disease	154	15.45	60	20.2	0.06
Chronic kidney disease	48	4.81	18	6.06	0.37
Malignancy before diagnosis	72	7.2	27	9.09	0.32

## WBC levels in patients with CS and controls

At baseline, the average WBC was 8.8 ± 2.88 Kcells/µl in patients with CS compared to 7.54 ± 2.59 Kcells/µl in controls (*p* < 0.0001), with leukocytosis present in 64 (21. 5%) of cases versus 89 (8.9%) controls (*p* < 0.0001). Patients with CS also had a higher mean neutrophil count (5.78 ± 2.43 Kcells/µl vs. 4.49 ± 1.89 Kcells/µl, *p* < 0.0001) and slightly lower mean lymphocyte count (2.12 ± 0.81 Kcells/µl vs. 2.24 ± 0.96 Kcells/µl, *p* = 0.014) than controls. The NLR was significantly elevated in patients with CS vs. controls (3.32 ± 2.45 Kcells/µl vs. 2.28 ± 1.75 Kcells/µl, *p* < 0.0001).

Two years post-surgery, patients with CS showed a persistently elevated WBC compared to controls (8.24 ± 2.33 Kcells/µl vs. 7.56 ± 2.36 Kcells/µl, *p* < 0.0001), with a notable decrease observed in patients with CS (−0.57 ± 2.6 Kcells/µl vs. 0.02 ± 1.98 Kcells/µl, *p* < 0.0001). Following surgery, 11.8% of patients with CS had leukocytosis compared to 7.7% of controls (*p* < 0.05), and 45 patients (70.3%) out of 64 with leukocytosis at baseline had normal WBC. Neutrophil counts remained higher in patients with CS (4.98 ± 2.18 Kcells/µl vs. 4.48 ± 1.94 Kcells/µl, *p* < 0.0001), with a greater decrease compared to controls (−0.84 ± 2.49 Kcells/µl vs. −0.02 ± 2.01 Kcells/µl, *p* < 0.0001). NLR showed a significant decrease observed in patients with CS (−0.63 ± 2.77 Kcells/µl vs. 0.06 ± 2.36 Kcells/µl, *p* < 0.0001) (Table 2).

**Table 2:** Blood count in all Cushing syndrome versus controls before and after surgery

	Control	All CS	*p*-value
**N**	997	297	
**%**	100	100	
**N**			
**WBC**			
2 years before diagnosis	7.54 ± 2.59	8.8 ± 2.88	< 0.0001
2 years post-surgery	7.56 ± 2.36	8.24 ± 2.32	< 0.0001
Absolute change	0.02 ± 1.98	−0.57 ± 2.56	0.0002
**Neutrophils**			
2 years before diagnosis	4.48 ± 1.97	5.82 ± 2.38	< 0.0001
2 years post-surgery	4.48 ± 1.94	4.98 ± 2.18	< 0.0001
Absolute change	0.02 ± 2.01	−0.84 ± 2.49	< 0.0001
**Lymphocytes**			
2 years before diagnosis	2.26 ± 1.02	2.09 ± 0.82	0.0057
2 years post-surgery	2.21 ± 0.99	2.22 ± 0.78	0.22
Absolute change	−0.06 ± 0.66	0.13 ± 0.81	< 0.0001
**NLR**			
2 years before diagnosis	2.27 ± 1.86	3.37 ± 2.63	< 0.0001
2 years post-surgery	2.33 ± 1.95	2.73 ± 2.67	0.06
Absolute change	0.06 ± 2.36	−0.63 ± 2.77	< 0.0001

(A) WBC, (B) neutrophils, (C) lymphocytes, (D) neutrophils to lymphocytes ratio

## WBC profile according to remission status

Data on remission status at 2 years was available for 253 patients with CS, with 178 achieving and 75 not achieving remission (Table 3).

**Table 3:** Blood count in cases with remission, no remission and controls

	Remission	Control	*p*-value	No remission	Control	*p*-value
**N**	178	615		75	253	
**%**	100	100		100	100	
**N**						
**WBC**						
2 years before diagnosis	8.56 ± 2.79	7.37 ± 2.34	< 0.0001	9.69 ± 3.16	7.84 ± 3.02	< 0.0001
2 years post-surgery	8.11 ± 2.40	7.46 ± 2.17	0.0004	8.71 ± 2.25	7.75 ± 2.70	0.0001
Absolute change	−0.46 ± 2.55	0.08 ± 1.74	0.0119	−1.01 ± 2.72	−0.09 ± 2.40	0.0039
**Neutrophils**						
2 years before diagnosis	5.68 ± 2.33	4.36 ± 1.87	< 0.0001	6.46 ± 2.42	4.67 ± 1.96	< 0.0001
2 years post-surgery	4.75 ± 2.22	4.47 ± 1.95	0.0336	5.60 ± 2.41	4.62 ± 2.04	0.0002
Absolute change	−0.96 ± 2.40	0.03 ± 1.90	< 0.0001	−0.86 ± 2.85	−0.09 ± 2.05	0.0165
**Lymphocytes**						
2 years before diagnosis	2.06 ± 0.82	2.22 ± 0.80	0.0153	2.17 ± 0.87	2.34 ± 1.46	0.2825
2 years post-surgery	2.21 ± 0.74	2.20 ± 0.92	0.3007	2.28 ± 0.85	2.30 ± 1.21	0.3566
Absolute change	0.16 ± 0.82	−0.03 ± 0.64	0.0008	0.11 ± 0.86	−0.04 ± 0.66	0.151
**NLR**						
2 years before diagnosis	3.32 ± 2.62	2.24 ± 1.97	< 0.0001	3.48 ± 2.00	2.32 ± 1.74	< 0.0001
2 years post-surgery	2.49 ± 1.97	2.30 ± 2.08	0.5211	3.02 ± 2.57	2.24 ± 1.23	0.0485
Absolute change	−0.81 ± 2.71	0.06 ± 2.60	< 0.0001	−0.44 ± 3.12	−0.10 ± 1.56	0.0089

(A) white blood cells, (B) neutrophils, (C) lymphocytes, (D) neutrophils to lymphocytes ratio

## Patients with CS who achieved remission

Patients with CS who achieved remission at 2 years post-surgery showed higher average WBC at baseline diagnosis compared to controls (8.56 ± 2.79 Kcells/µl vs. 7.37 ± 2.37 Kcells/µl, *p* < 0.0001). They also exhibited elevated mean neutrophil counts (5.68 ± 2.33 Kcells/µl vs. 4.36 ± 1.87 Kcells/µl, *p* < 0.0001) and slightly lower, mean lymphocyte counts than controls (2.06 ± 0.82 Kcells/µl vs. 2.22 ± 0.8 Kcells/µl *p* = 0.0153); The NLR was significantly higher (3.32 ± 2.62 vs. 2.24 ± 1.97, *p* < 0.0001).

Two years following surgery, those in remission still showed slightly elevated WBC counts compared to controls (8.11 ± 2.4 Kcells/µl vs. 7.46 ± 2.17 Kcells/µl, *p* = 0.0004), with a notable decrease observed over time in patients with CS (−0.46 ± 2.55 Kcells/µl) but not in controls (0.08 ± 1.74 Kcells/µl) (*p* = 0.0119). The neutrophil count was 4.71 ± 2.10 Kcells/µl in CS patients and 4.41 ± 1.87 Kcells/µl in controls (*p* = 0.0336). Lymphocyte count was 2.21 ± 0.74 Kcells/µl in patients with CS compared to 2.2 ± 0.92 Kcells/µl in controls (*p* = 0.3). The NLR at this point did not differ significantly between remission patients and controls.

### Patients with CS who did not achieve remission

At baseline, patients with CS who did not achieve remission exhibited higher average WBC (9.69 ± 3.16 Kcells/µl WBC vs. 7.84 ± 3.02 Kcells/µl, *p* < 0.0001). They also had higher mean neutrophil counts 6.46 ± 2.42 Kcells/µl vs. 4.67 ± 1.96 Kcells/µl, *p* < 0.0001), no difference in mean lymphocyte counts (2.17 ± 0.87 Kcells/µl vs. 2.34 ± 1.46 Kcells/µl, *p* = 0.28), and a higher NLR (3.48 ± 2.0 vs. 2.32 ± 1.74, (*p* < 0.0001), compared to controls.

Two years after surgery, patients with CS who did not achieve remission still had significantly higher WBC count compared to controls (8.71 ± 2.25 Kcells/µl vs. 7.75 ± 2.7 Kcells/µl, *p* = 0.0001). The absolute change in WBC was greater in these patients compared to controls (−1.01 ± 2.72 Kcells/µl vs. −0.09 ± 2.4 Kcells/µl, *p* = 0.0039). They also had higher neutrophil counts (5.6 ± 2.41 Kcells/µl vs. 4.62 ± 2.04 Kcells/µl, *p* = 0.0002). The NLR remained higher in patients with CS who did not achieve remission compared to controls (3.02 ± 2.57 vs. 2.24 ± 1.23, *p* = 0.048), with a greater absolute change in NLR (− 0.44 ± 3.12 vs. −0.1 ± 1.56, *p* = 0.0089).

## Baseline leukocytosis as a predictor of Non-remission

Among 253 patients with remission status data, 57 had baseline leukocytosis. Of these, 35 patients (61.4%) achieved remission, while 22 patients (38.5%) did not attain remission 24 months post-surgery. In comparison, of the 196 patients with normal baseline WBC counts, 143 (73.0%) achieved remission, and 53 (27.0%) did not. Although patients with baseline leukocytosis had a lower remission rate at 2 years compared to those with normal WBC (38.5% vs. 73.0%), the difference was not statistically significant (*p* = 0.09 h = 0.59, 95% CI 0.32–1.10).

In a subgroup of 113 patients with CD (for whom remission status data was available), 30 had baseline leukocytosis, while 83 had normal WBC. At 24 months post-surgery, remission rates were lower in patients with baseline leukocytosis compared to those with normal WBC levels (36.6% vs. 63.9%, respectively; *p* = 0.010).

In the subgroup analysis of adrenal CS, remission rates did not differ based on baseline leukocytosis status.

## WBC Profile according to Disease Etiology

Etiology was available for 246 patients with CS, comprising 119 patients with CD and 127 patients with adrenal CS (aCS). These patients were matched with 373 and 433 controls, respectively. (Table 4)

**Table 4:** Blood count in cases with adrenal Cushing syndrome, Cushing disease and controls before and after surgery. (A) white blood cells, (B) neutrophils, (C) lymphocytes, (D) neutrophils to lymphocytes ratio table 1

	Adrenal	Control	*p*-value	Pituitary	Control	*p*-value
**N**	127	433		119	373	
**%**	100	100		100	100	
**N**						
**WBC**						
24 months before diagnosis	8.78 ± 2.97	7.47 ± 2.67	< 0.0001	9.23 ± 2.94	7.69 ± 2.61	< 0.0001
24 months post-surgery	8.17 ± 2.23	7.5 ± 2.41	0.0004	8.55 ± 2.38	7.73 ± 2.42	0.0001
Absolute change	−0.63 ± 2.6	0.03 ± 1.99	0.0157	−0.69 ± 2.62	0.05 ± 2.09	0.0055
**Neutrophils**						
24 months before diagnosis	5.87 ± 2.54	4.38 ± 1.92	< 0.0001	6.2 ± 2.35	4.61 ± 2.02	< 0.0001
24 months post-surgery	4.59 ± 1.77	4.38 ± 1.94	0.1376	5.43 ± 2.51	4.6 ± 2.01	0.0002
Absolute change	−1.27 ± 2.37	−0.03 ± 2.07	< 0.0001	−0.76 ± 2.57	−0.02 ± 2.15	0.0015
**Lymphocytes**						
24 months before diagnosis	2.10 ± 0.79	2.29 ± 1.28	0.1216	2.03 ± 0.89	2.24 ± 0.78	0.0039
24 months post-surgery	2.19 ± 0.66	2.24 ± 1.21	0.4622	2.26 ± 0.84	2.23 ± 0.79	0.3938
Absolute change	0.09 ± 0.83	−0.05 ± 0.65	0.0286	0.23 ± 0.78	−0.03 ± 0.69	0.0008
**NLR**						
24 months before diagnosis	3.19 ± 1.85	2.20 ± 1.48	< 0.0001	3.96 ± 3.48	2.39 ± 2.46	< 0.0001
24 months post-surgery	2.31 ± 1.29	2.33 ± 2.29	0.0001	3.2 ± 3.69	2.33 ± 1.79	0.0324
Absolute change	−0.87 ± 2.03	0.12 ± 2.3	< 0.0001	−0.73 ± 3.41	−0.05 ± 0.81	< 0.0001

### Patients with adrenal CS

At baseline, patients with aCS exhibited higher WBC counts and neutrophil counts compared to controls (*p* < 0.0001), with no significant difference in lymphocyte counts. NLR was significantly elevated in aCS patients (3.19 ± 1.85 vs. 2.20 ± 1.48 in controls; *p* < 0.0001). Two years post-surgery, aCS patients showed a decrease in WBC (−0.63 ± 2.48 Kcells/µl; *p* = 0.015) and neutrophil counts (−1.27 ± 2.37 Kcells/µl; *p* < 0.0001) compared to controls, while lymphocyte counts and NLR did not differ between patients with aCS and controls at this point.

### Patients with CD

At baseline, patients with CD had elevated WBC (9.23 ± 2.94 Kcells/µl vs. 7.69 ± 2.61 Kcells/µl, *p* < 0.0001) and neutrophil counts (6.2 ± 2.35 Kcells/µl vs. 4.61 ± 2.02 Kcells/µl, *p* < 0.0001) with lower lymphocyte counts (2.03 ± 0.89 Kcells/µl vs. 2.24 ± 0.78 Kcells/µl, *p* = 0.0039) compared to controls. NLR was significantly higher in CD (3.96 ± 3.48 vs. 2.39 ± 2.46; *p* < 0.0001). Two years after surgery, CD patients experienced significant reductions in WBC − 0.69 ± 2.62 Kcells/µl; *p* = 0.0055), neutrophil − 0.76 ± 2.57 Kcells/µl; *p* = 0.0015), and NLR (−0.73 ± 3.41; *p* < 0.0001), while lymphocyte counts increased slightly compared to controls.

## Discussion

In our study, we have shown that WBC profile could hold both diagnostic and prognostic value in patients with CS. From a diagnostic perspective, patients with CS exhibit significantly higher average WBC counts compared to sex-, BMI and socioeconomic-matched controls, with notably higher rates of leukocytosis. This suggests that CS should be considered in the workup for patients with unexplained leukocytosis. Prognostically, our data indicate that baseline leukocytosis in patients with CD may be associated with lower remission rates compared to those with normal baseline WBC counts, highlighting WBC as a potential predictor of remission likelihood.

Although excluding patients with CS missing surgical data revealed a three-year difference, this disparity does not influence WBC count, as demonstrated in a previous study comparing young and old individuals [16]. Matching to BMI was important due to several studies reporting WBC correlates with BMI rise [17, 18]. Women are reported to have higher WBC, in our study matching was also for sex [19, 20].

Though found in approximately 20% of patients with CD, we consider that if present at baseline, leukocytosis could be valuable in preoperative assessments alongside other factors like cavernous sinus invasion, tumor size, preoperative ACTH levels, and disease duration, all of which have been linked to higher chance of immediate remission [3, 21]. This correlation, however, was not observed in patients with aCS, indicating a different disease progression history.

Additionally, patients with CS showed a significant reduction in mean WBC, neutrophil counts, and NLR post surgery compared to controls, irrespective of remission status, suggesting a possible link to disease severity.

Cortisol exerts its effects through binding to the glucocorticoid receptor [22]. Administration of cortisol has been shown to induce various alterations in blood count, most notably leukocytosis and neutrophilia [23–26]. Despite this increase, the inflammatory function of these cells is downregulated, resulting in heightened susceptibility to infections [22, 27]. The rise in cortisol levels leads to an increased number of circulating granulocytes by enhancing their release from the bone marrow into circulation while simultaneously reducing their egress from the granulocyte pool [28].

Our previous research on a small cohort of patients with CD demonstrated that only 40% had leukocytosis, with WBC counts significantly lower in those who achieved remission compared to levels before diagnosis [12]. Another study of 73 patients with CS compared blood parameters before and after therapy, albeit without controls, yielding similar findings [29]. In the current study, 20% of patients with CS had leukocytosis at baseline, with 70% normalizing their leukocyte levels post-surgery. Additionally, the average WBC count for all patients decreased two years after surgery, even among those who did not achieve remission, aligning with findings that correlate WBC levels with cortisol concentrations [10].

Lymphopenia is a characteristic feature of CS, contributing to fatal infections due to impaired late and acquired immunity [27, 30]. This observation in our data is consistent with previous studies [10–12]. Detomas et al. reported a continuous rise in lymphocyte levels at 3, 12, and 24 months after remission in patients with CD, but this pattern was not observed in those with aCS, and data on the non-remission group were not provided [10]. In contrast to WBC and neutrophils, which were higher in the non-remission group than in controls in our study, post-surgical lymphocyte counts remained comparable to controls, irrespective of remission status.

Whether this normalization of lymphocyte counts translates to a reduced infection risk post-treatment requires further study. Additionally, the comparable lymphocyte counts in aCS patients before and after surgery compared to controls may explain the lower infection rates in this group [31], but this too warrants further investigation.

The NLR has recently gained attention as a marker of inflammation, complications, and mortality across various conditions [32]. Studies have shown a correlation between NLR and cortisol levels in patients with adrenal incidentalomas [33], and higher NLR in pediatric patients with CS compared to controls [13]. In the current study, NLR was elevated in all CS groups relative to controls, and this trend persisted post-treatment in the non-remission patients. Specifically, patients with CD continued to show elevated NLR 2 years after surgery, whereas aCS patients had NLR values comparable to controls at the same time point. The persistent elevation in NLR is linked to an increased mortality risk—up to 5.6 times higher in persistent CD compared to matched controls, and a 1.5-fold increase in patients in remission [34]. Further research is needed to determine if a specific NLR threshold correlates with increased mortality in CS.

Similar trends in WBC profiles were observed in both aCS and CD, aligning with previous studies on WBC profiles in CS patients [9, 10, 29, 35]. These findings reflect the impact of cortisol on WBC counts through various mechanisms, a similar effect to that seen following exogenous glucocorticoid administration [24, 26]. The time to diagnosis in both adrenal CS and CD often exceeds 30 months, typically based on clinical suspicion or patient-reported symptoms [36]. Early diagnosis of CS is crucial for reducing morbidity and mortality [37], underscoring the need to incorporate more clinical and laboratory markers for diagnosis after an early suspicion of CS. Paja et al. reported that an NLR greater than 2.9 had a sensitivity of 78.1% and a specificity 88.4% for diagnosing CS in a cohort of 76 patients with different etiologies, including ectopic CS, compared to controls with non-secreting adrenal adenomas [9]. However, further investigation is necessary to validate this threshold in larger cohorts compared to the general population.

This study represents the largest analysis of WBC differentiation and changes before and after surgery in patients with CS. However, several limitations should be noted. This was a retrospective database study relying on the coding of diagnosis dates, though a stringent review of laboratory values helped refine the patient cohort; on the other hand we likely missed cases due to the strict diagnosis criteria, and the results may not apply to milder cases. Remission status was defined only by UFC results due to inability to retrieve cortisol suppression test results from the database, moreover salivary cortisol was not incorporated yet in CHS in the years of the study. There was no standardized protocol for blood test intervals, and the reasons for performing these tests, particularly in the control group, were not available. Additionally, patients with a confirmed diagnosis but lacking surgery dates or WBC counts were excluded from the analysis.

In conclusion, our study demonstrates that patients with CS exhibit significant alterations in blood count components before diagnosis. Leukocytosis, found in one out of five CS patients, may serve as a negative preoperative predictor of remission in CD patients but not in aCS patients. While some blood count abnormalities diminish after surgery, neutrophil counts remain elevated, and NLR continues to be higher in patients with CD post-treatment. The correlation between leukocytosis and remission rates in patients with CD, as well as the relationship between NLR and complication rates following treatment, warrants further investigation.

## Abbreviations

CS: Cushing’s syndrome
BMI: Body mass index
DM: Diabetes mellitus
HTN: Hypertension
IHD: Ischemic heart disease
CD: Cushing’s disease
aCS: adrenal Cushing’s syndrome
